# *In Vitro* and *In Vivo* Hepatic Differentiation of Adult Somatic Stem Cells and Extraembryonic Stem Cells for Treating End Stage Liver Diseases

**DOI:** 10.1155/2015/871972

**Published:** 2015-08-05

**Authors:** Chenxia Hu, Lanjuan Li

**Affiliations:** Collaborative Innovation Center for Diagnosis and Treatment of Infectious Diseases, State Key Laboratory for Diagnosis and Treatment of Infectious Diseases, School of Medicine, First Affiliated Hospital, Zhejiang University, Hangzhou, Zhejiang ZJ 571, China

## Abstract

The shortage of liver donors is a major handicap that prevents most patients from receiving liver transplantation and places them on a waiting list for donated liver tissue. Then, primary hepatocyte transplantation and bioartificial livers have emerged as two alternative treatments for these often fatal diseases. However, another problem has emerged. Functional hepatocytes for liver regeneration are in short supply, and they will dedifferentiate immediately *in vitro* after they are isolated from liver tissue. Alternative stem-cell-based therapeutic strategies, including hepatic stem cells (HSCs), embryonic stem cells (ESCs), induced pluripotent stem cells (iPSCs), and mesenchymal stem cells (MSCs), are more promising, and more attention has been devoted to these approaches because of the high potency and proliferation ability of the cells. This review will focus on the general characteristics and the progress in hepatic differentiation of adult somatic stem cells and extraembryonic stem cells *in vitro* and *in vivo* for the treatment of end stage liver diseases. The hepatic differentiation of stem cells would offer an ideal and promising source for cell therapy and tissue engineering for treating liver diseases.

## 1. Introduction

Viral infections, toxic injury, autoimmune disorders, or genetic disorders may cause severe liver dysfunction and result in acute liver failure (ALF) or chronic liver disease, which has become the most important cause of morbidity and mortality in the world. Once liver transplantation has been the sole method to treat terminal liver failure, it is limited by numerous problems, including a chronic shortage, high cost, immune rejection, and side effects. Next, cell transplantation and artificial livers emerged as two effective alternative therapeutic approaches. No matter which method is selected, it is urgent for patients to receive sufficient functional hepatocytes for liver regeneration. However, human primary hepatocytes are scarce in number, have limited proliferation potential, and have rapid phenotypic dedifferentiation* in vitro*. After hepatocytes are isolated, they need abundant and constant supplies of oxygen and nutrients to maintain their viability and phenotype and to perform unique liver functions (e.g., detoxification, synthesis of numerous factors, and the regulation of metabolism). To solve these problems, stem-cell-based therapeutic strategies, including hepatic stem cells (HSCs), embryonic stem cells (ESCs), induced pluripotent stem cells (iPSCs), and mesenchymal stem cells (MSCs), have emerged as alternative options. In this study, the term MSCs refers to adult somatic stem cells and extraembryonic stem cells, which are derived from adult organs (except liver tissue) and extraembryonic tissue. These cells have more potency and organ availability compared to HSCs, no ethical issues compared to ESCs, and less tumorigenicity compared to iPSCs. From this perspective, adult somatic stem cells and extraembryonic stem cells are more suitable for patients and lead to fewer negative side effects.

The long-term self-renewal capability, long-term stability in* in vitro* culture, the differentiation capacity, and the low immune rejection of stem cells have prompted scientists to concentrate on the* in vitro* and* in vivo* differentiation of stem cells into endodermal and ectodermal lineages. To standardize MSCs, the International Society for Cell Therapy suggested the following minimal criteria [1]: plastic adherence in conjunction with a fibroblastoid phenotype; expression of CD105, CD73, and CD90, and lack of expression of CD45, CD34, CD14 (or CD11b), CD79*α* (or CD19), and HLA-DR surface molecules; differentiation capacity towards chondrocyte, adipocyte, and osteocyte lineages. Even with these standards, different sources of MSCs have varied in their surface markers and differentiation disposition.

The hepatic differentiation potential of MSCs may provide an unlimited source of cells for hepatocyte replacement therapies [2, 3]. To ensure the hepatic therapeutic success of these cells, characteristics such as* in vitro* expandability, the expression of hepatic like surface markers, hepatic cell function, and minimal or absent immunogenicity in the recipient host need to be considered [4]. To date, four primary strategies have been developed to induce MSCs into hepatocytes: the addition of chemical compounds and cytokines, genetic modification, adjustment of the microenvironment, and alteration of the physical parameters used for culturing MSCs. On the other hand, obtaining the same hepatic functions as primary hepatocytes* in vivo* is hard to achieve, which has hindered the clinical usage of these cells for treating end stage liver diseases. In this review, we primarily investigated adult somatic stem cells and extraembryonic stem cells originating from various tissues, which have been widely investigated in regenerative medicine applications. This study presents the current knowledge regarding the general characteristics of these cells, as well as the differentiation of these cells into functional hepatocytes* in vitro* and* in vivo*. Then, the paracrine effects of them were also discussed for investigating the* in vivo* mechanism of repairing injured liver tissue.* In vitro* and* in vivo* differentiation of stem cells into mature and functional hepatocytes may offer an ideal and promising source for cell therapy and tissue engineering for treating liver diseases.

## 2. Adult Somatic Stem Cells

### 2.1. Main Sources of Adult Somatic Stem Cells

Adult somatic stem cells are isolated from adult somatic tissue, and they are fibroblast-like, nonhematopoietic, and plastic-adherent cells. Though adult somatic stem cells can be isolated from anywhere in the human body, there are several main sources for hepatic differentiation when cell number and convenience are considered.

In 1970, Friedenstein et al. [5] first reported the isolation of bone marrow mesenchymal stem cells (BMMSCs) with density gradient centrifugation, and their approach was widely studied from that point on. Adult bone marrow aspirates of the os coxae after puncture of the crista iliaca, or surgical waste gained from amputations or knee and hip operations, are an abundant supply of stem cells for regenerative medicine. Then, in 2001, Zuk et al. [6] for the first time isolated adipose derived mesenchymal stem cells (ADMSCs) by collagenase. ADMSCs are abundant and accessible, not only because they are easy to obtain from liposuction aspirates or excised fat [7] but also because of their immunosuppressive properties. Another primary type of adult somatic stem cells, menstrual blood stem cells (MenSCs), is a type of mononuclear cells derived from the endometrium, and it was first derived in 2007 [8]. These cells exhibited mesenchymal surface markers, including CD56, CD73, CD90, CD105, and CD146 in parallel to the embryonic markers OCT-4 and SSEA-4 [9]. The advantages of MenSCs, such as easy access, minimal ethical considerations, and high proliferative ability, have inspired scientists to investigate the potential of cell therapy for different diseases [8].

### 2.2. *In Vitro* Hepatic Differentiation of Adult Stem Cells

In the past decade, several hepatic differentiation systems of BMMSCs have been established. Using combined exposure to growth factors and nutrients, such as fibroblast growth factor (FGF), hepatocyte growth factor (HGF), insulin-transferrin-selenium, and dexamethasone, these cells will transform into cells with the morphological, phenotypic, and functional characteristics of hepatocytes [10]. The addition of insulin growth factor 1, nicotinamide and hepatocyte nuclear factor (HNF)-4*α*, and valproic acid [11] can significantly improve the hepatic differentiation rate of human BMMSCs, as well as enhancing the expression level of hepatic specific genes. Lu et al. [12] demonstrated these characteristics after seven days of induction with HGF and FGF-4 followed by incubation with inhibitors p38, ERK1/2, and MSK1 in the differentiation medium. As a result, the expressions of alpha fetoprotein (AFP) and FOXa2 in the p38 inhibitor group were reduced compared to the ERK1/2 inhibitor group. Next, the researchers concluded that the mitogen-activated protein kinase cell-signaling pathway may drive the differentiation of mouse BMMSCs into hepatocytes. Because an* in vivo* environment for cell development may contain a different supporting medium,* in vitro* culture (which is analogous to and* in vivo* environment) may promote the differentiation of BMMSCs. These properties were clarified by reports about three-dimensional alginate scaffolds [13], collagen-coated polyscaffolds [14], dynamic cultured scaffolds [15], and nanofibers [16]. All of these scaffolds are able to introduce a better differentiation of BMMSCs that leads the cells to exhibit the ultrastructural characteristics of mature hepatocytes, as well as to express endodermal and hepatocyte specific genes and proteins associated with improved hepatic function. According to the latest research, a decellularization process preserved the fibrillar microstructure and a mix of matrix proteins in a cell-deposited extracellular matrix in native liver, and then the hepatocyte-like cells (HLCs) differentiated from BMMSCs on extracellular matrix were identified by more intense glycogen storage staining, an elevated level of urea biosynthesis, and higher expressions of hepatocyte specific genes compared to tissue culture polystyrene [17].

Previous studies have shown that ADMSCs can differentiate into hepatocytes when cultured with various cytokines and growth factors associated with liver regeneration [18], such as interleukin- (IL-) 6, HGF, vascular endothelial growth factor (VEGF) [19–22], and dimethyl sulfoxide [23]. Presumably, due to their mesenchymal lineage, human ADMSCs do not express endodermal transcription factors, such as Foxa1, Foxa2, and Gata4. However, supplementation of these transcription factors may induce competency and enhance the differentiation of human ADMSCs into hepatocytes [24]. Epigenetic modification will regulate the differentiation effects of stem cells and induce them to obtain more mature functions for liver regeneration. Alizadeh et al. [25] showed that transient inhibition of liver enriched transcription 7 microRNAs activates the hepatic differentiation of human ADMSCs, and overexpression of miR-122 in ADMSCs resulted in the increased expression of specific hepatocyte markers. Urea and albumin (ALB) production as well as glycogen deposits demonstrated the same trend [26]. Various extracellular matrix components were employed as coating materials to promote hepatic differentiation from ADMSCs, even though no consensus was achieved about the optimal coating matrix. Chitosan hydrogel [27] promotes cell proliferation coupled with >90% cell viability, and glutaraldehyde cross-linked chitosan exhibited <5% cytotoxicity. Thus, the chitosan served as a scaffold and facilitated the expansion and differentiation of ADMSCs across endoderm, ectoderm, and mesoderm lineages. Culturing human ADMSCs on top of HGF/Col spots (HGF coprinted with collagen I to create arrays of protein spots on glass) for 2 weeks can induce differentiation into HLCs [28]. Next, a direct comparison between several coating extracellular matrices was performed for the hepatic differentiation of ADMSCs [29]. They demonstrated that liver decellularized liver matrix as a coating matrix could significantly enhance the hepatic differentiation of ADMSCs compared to collagen, fibronectin, and Matrigel in both the presence and the absence of growth factors. The differentiated cells would enhance hepatocyte specific genes expression as well as hepatocyte related protein secretion with improved liver functions.

MenSCs are also able to differentiate into functional HLCs* in vitro*. After three weeks of incubation in hepatic differentiation medium containing HGF, FGF-4, and oncostatin M, cuboidal cells were observed, and these cells also expressed hepatocyte specific marker genes. The differentiated cells demonstrated mature hepatocyte functions* in vitro*, such as urea synthesis, glycogen storage, and indocyanine green uptake [30]. Another study regarding the MenSCs derived HLCs functional hepatocyte markers at the mRNA and protein levels demonstrated that GSTA1, GSTA2, and cytochrome (CYP) 3A4 mRNA were upregulated in differentiated cells compared to undifferentiated cells. However, the expression of the CYP7A1 gene was remarkable until the last day of the differentiation process [31]. The degree of MenSCs hepatic differentiation depended on the concentrations of cytokines, and the omission of serum during the process led to improvement in hepatocyte specific functions. The levels of ALB and CYP7A1 were higher in differentiated MenSCs compared to driven BMMSCs; however, the level of CK18 and ALB, and the glycogen accumulation were lower or not significantly different compared to the controls [32].

### 2.3. *In Vivo* Hepatic Differentiation of Adult Stem Cells

Intrasplenic transplantation into the carbon tetrachloride (CCl_4_) injured livers of SCID mice enhanced human BMMSCs grafted into the host liver parenchyma, exhibited typical hepatocyte morphology, and formed a three-dimensional architecture, and the engrafted BMMSCs differentiated into HLCs* in vivo* [33]. Li et al. [34] showed that autologous BMMSCs can differentiate into hepatocytes and promote future liver remnant regeneration after portal vein embolization in cirrhotic liver, which may improve the local microenvironment by decreasing cirrhosis, upregulating the gene expression of VEGF, HGF, IL-10, and matrix metalloproteinase 9. Undifferentiated BMMSCs transplantation can promote liver regeneration and BMMSCs derived HLCs transplantation can also repair injured liver. After the cells were differentiated into HLCs* in vitro* and then transplanted into the livers of immunodeficient mice with acute liver injuries, clusters of transplanted cells appeared predominantly in the periportal portion of the liver lobule and secreted human ALB in addition to featuring prominent qualities of differentiated hepatocytes [35]. Amer et al. [36] demonstrated that autologous BMMSCs derived hepatocytes transplantation in patients with end stage liver cell failure showed statistically significant improvement in child score, model for end stage liver disease score, fatigue scale, and performance status over the traditional supportive treatment. Then, Li et al. [37] compared BMMSCs and induced BMMSCs transplantation efficacy for liver failure and found that they had a similarly positively therapeutic efficacy in rat liver failure model. To improve the effect of transplantation, BMMSCs derived HLCs were pretreated with a combination of dynamic cultured scaffold and growth factors and transplanted into CCl_4_ injured mice, which increased their survival rate, liver function, engraftment into the host liver, and further hepatic differentiation [15].

Transplanted ADMSCs, as well as BMMSCs, are able to improve liver functions and promote liver regeneration [38], and these cells have exhibited the potential to differentiate into HLCs in the injured livers* in vivo *[39]. Because the transplantation effect was not maximized, a method was needed to increase the survival rate. In a rat liver injury model, intravenously injected ADMSCs successfully engrafted into recipient livers and the injection via the hepatic portal vein was more efficient than via the dorsal vein of the penis [40]. Intriguingly, successfully transplanted ADMSCs in liver tissue modulated kupffer cell activity to inhibit tumor necrosis factor- (TNF-) *α* secretion and this improved ADMSCs transplantation efficiency and therapeutic potential in liver injuries [41]. Then, Zhang et al. [42] showed that spheroid derived ADMSCs showed more effective potentials to rescue liver failure than ADMSCs derived from constant monolayer culture.

BMMSCs and ADMSCs have been widely studied in recent years to determine their* in vitro* and* in vivo* effects for treating liver diseases. The effects of MenSCs were not clarified that much and this lack of clarification may contribute to the new interest in MenSCs development. After intrasplenic transplantation into mice with a 2/3 partial hepatectomy, MenSCs derived HLCs were detected in the recipient livers and the cells expressed human ALB protein, restored the serum ALB level, and significantly suppressed transaminase activity in the animals with liver injuries [30].

## 3. Other Types of Adult Stem Cells

Epithelial stem cells from the rat pancreas were isolated and transplanted into the liver of an inbred strain of Fischer rats. The stem cells differentiated into hepatocytes, expressed liver specific proteins, and became fully integrated into the liver parenchymal structure [43]. To the best of our knowledge, the identity of pancreatic stem cells is still under debate. Rat pancreatic stellate cells, which reside in islets and between acini, display a gene expression pattern similar to BMMSCs. Cytokine treatments induced the expression of typical hepatocyte markers and the expression of endodermal proteins such as the bile salt export pump protein along with the mesodermal protein vimentin [44]. The transplantation of* in vitro* culture activated pancreatic stem cells from enhanced green fluorescent protein (EGFP) expressing rats into wild type rats in the presence of 2-acetylaminofluorene after the rats received a partial hepatectomy revealed that pancreatic stem cells were able to reconstitute large areas of the host liver through differentiation into hepatocytes and cholangiocytes [44].

The synovial membrane is a specialized mesenchymal tissue lining the spaces of diarthrodial joints, bursae, and tendon sheaths [45]. The synovial membrane includes two layers: the intima inner layer, which is composed of one or two sheets of macrophages or fibroblast-like synoviocytes, and the subintima outer layer, which is composed of two to three layers of synoviocytes lying over loose connective tissue rich in fibroblasts, secreting collagen, and other extracellular matrix proteins. When surface epitopes and proliferation potentialities are considered, synovium derived MSCs are similar to the BMMSCs and have a greater chondrogenesis potentiality. Therefore, whether synovium derived MSCs can be induced into HLCs needs to be studied further [46].

Park et al. [47] isolated cells from human dermis, which they called human dermis derived MSCs. The cells possessed the capacity to differentiate into multiple lineages, including adipocyte, osteocyte, and chondrocyte lineages as well as the precursor of a hepatocyte lineage.

## 4. Extraembryonic Stem Cells

### 4.1. Sources of Extraembryonic Stem Cells

Extraembryonic stem cells can be isolated from the placenta, umbilical cord, umbilical cord blood, and amniotic fluid. These cells are all derived from a pregnant woman or infant, but, due to ethical considerations, the cells are not collected from embryonic tissue. Placental derived mesenchymal stem cells (PDMSCs) might be an easily accessible source of ESCs because these cells reside in the fetal membranes of the term placenta, can be accessed noninvasively, and have high proliferative potential, a short population doubling time, and no ethical concerns about the collection of PDMSCs [48, 49]. Umbilical cord mesenchymal stem cells (UCMSCs) are single cell derived, clonally expanded MSCs isolated from discarded extraembryonic tissue after birth through the subendothelial layer of the human umbilical cord vein. These cells have a multilineage differentiation potential including a substantial hepatic differentiation capacity [50, 51]. They have been recognized as an ideal cell source for clinical use because of the painless collection, easy procurement, abundant availability, faster self-renewal, lower risk of viral contamination and tumor formation, and low immunogenicity [52]. Umbilical cord blood remains in the placenta and the umbilical cord after birth. These tissues were usually discarded after delivery as medical waste. However, since the presence of hematopoietic stem cells in umbilical cord blood was first discovered in 1974, the mononuclear cells were defined as umbilical cord blood mesenchymal stem cells (UCBMSCs). The extensive characterization of UCBMSCs has revealed that they are similar to BMMSCs not only with respect to their cellular properties and multilineage differentiation potential but also with respect to the molecular context of the cells [53, 54]. For cell procurement and transplantation, umbilical cord blood has many advantages over bone marrow, such as vast abundance, lack of donor attrition, low risk of viral transmission, and a less pronounced immune response. The fact that certain progenitor cells are found in the amniotic fluid was first reported in 1993 when small, nucleated, round cells identified as stem cells were found before the 12th week of gestation [55]. Furthermore, in 2003 Prusa et al. [56] isolated OCT4 positive amniotic fluid cells that expressed the stem cell factors vimentin and alkaline phosphatase as well as cyclin A mRNA. These cells are routinely obtained with minimally invasive techniques for prenatal diagnosis of fetal abnormalities. The usage of these cells is widespread and well established in prenatal genetic testing.

### 4.2. *In Vitro* Hepatic Differentiation of Extraembryonic Stem Cells

Lee et al. [57] declared that the proliferative potential of PDMSCs and the expression of hepatocyte markers in differentiated PDMSCs were higher than other MSCs (BMMSCs, ADMSCs, and UCMSCs). These cells are capable of differentiating into HLCs* in vitro* [58], and the differentiated cells gain hepatocyte-like morphologies, express hepatocyte specific markers, uptake lipoprotein, and store glycogen. The addition of rifampicin also increased expression of CYP3A4, which is similar to the activity of human liver cells [59]. PDMSCs contain several types of stem cells based on placental anatomy: chorionic villi mesenchymal stem cells (CV-MSCs), amnion mesenchymal stem cells (AE-MSCs), and chorionic plate mesenchymal stem cells (CP-MSCs) [60]. Many studies focus on a particular type of PDMSCs and their hepatic differentiation. Stem cell factor expression in CP-MSCs is significantly higher than other PDMSCs. The administration of CP-MSCs promoted liver repair through systemically concomitant mechanisms involving HIF-1*α* and autophagy, which decreased the necrotic cells and increased autophagic signals observed in hepatocytes during* in vitro* coculture with CP-MSCs [60]. AE-MSCs possess the ability to differentiate into cells with characteristics of functional HLCs [61, 62]. AE-MSCs derived HLCs can be encapsulated within alginate microcapsules without losing their viability or function* in vitro*. Furthermore, CYP3A4 activity and urea synthesis from the encapsulated HLCs were higher compared to HLCs in a monolayer culture environment [62]. When coculturing the subtype CD200 positive CV-MSCs with hepatocytes at ratios of 1 : 1 and 3 : 1, urea synthesis, ALB secretion, and hepatocyte proliferation were enhanced significantly. In addition, CV-MSCs inhibit hepatocyte apoptosis via upregulation of antiapoptotic protein [63].

UCMSCs were cultured under prohepatogenic conditions similar to conditions used for BMMSCs and differentiated following the induction of HGF and FGF-4 [64]. The absence of some hepatic markers (HepPar1 or hepatocyte nuclear factor 4) implied that differentiated UCMSCs did not reach the level of mature hepatocytes and they partially preserve MSC markers [65]. MSCs derived from the main component of the umbilical cord extracellular matrix are called Wharton's jelly MSCs (WJ-MSCs) [66], and then a simple, highefficiency, and time-saving method was developed to induce WJ-MSCs cell lines into a hepatic lineage within 18 days under hypoxic conditions [67]. Because the cell-cell interaction may promote the differentiation rate, contact or noncontact coculture with rat hepatocytes improved ALB secretion and urea genesis maintenance of human UCMSCs compared to monoculture [68]. Epigenetic modification also effectively encouraged the hepatic differentiation of UCMSCs. A histone deacetylase inhibitor, valproic acid, induced an increase in the expression of endodermal genes in human UCMSCs via the signal transduction of AKT and ERK activation [69].

A comparison between UCBMSCs and BMMSCs demonstrated that some hepatic markers were expressed in both types of cells. But UCBMSCs also expressed proliferating cell nuclear antigen, which implies that hepatocyte lineage cells have proliferative potential. Both types of cells can give rise to HLCs under simple culture conditions with cytokines [70]. UCBMSCs [71] were morphologically transformed into HLCs, and they expressed Thy-1, c-Kit, and Flt-3 at the cell surface as well as ALB and AFP, and CK18, and CK19 in the interior. Umbilical cord blood cells might represent a novel subpopulation of cord blood derived stem cells capable of successful differentiation into HLCs. When incubated with CFSC/HGF cells, *β* expressed several hepatocyte specific genes, and the cells also displayed the liver specific functions of ammonium metabolism and ALB secretion. However, the mechanism involved in these changes has yet to be determined. Downregulated expression of Wnt/beta-catenin-related genes and the translocation of beta-catenin were observed along the cell membrane and in the cytoplasm, although some beta-catenin was still in the nucleus [72]. Downregulation of Wnt/beta-catenin signals in the cells by Fz8-small interference RNA treatment resulted in similar hepatic differentiation to that observed with cytokines. In addition, the subcellular distribution of beta-catenin was similar to that of cells treated with cytokines.

Amniotic fluid mesenchymal stem cells (AF-MSCs) are as genetically stable as BMMSCs, and they showed a higher hepatic differentiation potential than BMMSCs [73]. After treatment with cytokines, AF-MSCs developed the morphology similar to hepatocytes and expressed the hepatocyte specific markers. At the protein level, the differentiated cells developed hepatocyte specific functions [74]. According to the latest study about AF-MSCs hepatic differentiation, canine AF-MSCs at passage 5 had a fibroblast-like morphology instead of forming colonies and were positive for pluripotent stem cell markers such as OCT4, NANOG, and SOX2 [75]. The cells still exhibited a fibroblast-like morphology after hepatic induction.

### 4.3. *In Vivo* Hepatic Differentiation of Extraembryonic Mesenchymal Stem Cells

After transplantation into the liver of retrorsine treated SCID/beige mice, naive human AE-MSCs differentiated into HLCs that expressed mature liver genes at levels equal to adult liver tissue [62]. Human AE-MSCs transplanted into immunocompetent mice gave rise to cell engraftment, reduced hepatocyte apoptosis, and decreased hepatic inflammation and fibrosis [76]. The potential pathophysiological roles of CP-MSCs include their antifibrotic effects in repairing liver function [77]. The expression levels of a smooth muscle actin and collagen I were lower in PKH26-labeled CP-MSCs transplanted rats, whereas the expression levels of ALB and the uptake of indocyanine green increased in transplanted rats. Cao et al. [78] confirmed that human PDMSCs could not only differentiate into HLCs* in vitro* and* in vivo*, but can also prolong the survival time of ALF pigs. The effect of the left branch of the portal vein transplantation was superior to the jugular vein pathway in treating acute liver failure.

*In vivo* investigations showed that, following systemic administration, UCMSCs are able to accelerate the resolution of an acute liver injury without any differentiation and manipulation [79]. UCMSCs can significantly improve the survival of rats with acute hepatic necrosis [80] and liver fibrosis [81]. The mechanisms underlying these improvements may include the reduction of hepatocyte denaturation, inhibition of hepatocyte apoptosis, decreasing serum aminotransferases, and facilitating hepatocyte proliferation [82]. Then, UCMSCs derived HLCs transplantation can take effect as well as undifferentiated one. When the cells are transplanted into mice with CCl_4_ induced liver injury, HLCs can not only improve liver function but also restore injured livers [83]. Zhou et al. [84] demonstrated that the therapeutic effects of human UCMSCs were mediated largely via the stimulation of host hepatocyte regeneration when the cells were delivered through intravenous injection. Poly(3-hydroxybutyrate-co-3-hydroxyvalerate-co-3-hydroxyhexanoate) scaffolds loaded with UCMSCs or differentiated UCMSCs had the similar effect on injured livers and significantly promoted the recovery of injured livers [85].

Tang et al. [86] showed that after a one-month treatment with human UCBMSCs, the rats with ALF were positive for human AFP and ALB in their liver tissue. The DNA fragment of the human X chromosome could be found in the rat liver tissue of group treated with human UCBMSCs. Shi et al. [80] also demonstrated that rats with acute hepatic necrosis that were transplanted with green fluorescent protein labeled human UCBMSCs had significantly lower death rates after 48 hours compared to the rats receiving no human UCBMSCs [87]. Human hepatocyte specific markers were detected in the rat liver tissue after 10 days of UCBMSCs infusion. Moreover, the upregulation of APE1 expression suggested that maintaining a high level of APE1 had protective effect by acting as a prosurvival signal. UCBMSCs were as effective as the UCBMSCs derived HLCs in ALF models, and the therapeutic effects of UCBMSCs were mediated largely via stimulation of host hepatocyte regeneration, though the populations of the UCBMSCs derived cells were small [64].

When the AF-MSCs were transplanted into CCl_4_ injured, immune-deficient mice, undifferentiated AF-MSCs were integrated into the liver tissue, and they expressed markers characteristic of mature human hepatocytes. Although the integration of AF-MSCs into the liver was limited (0.1–0.3% of hepatocytes), histological analysis showed that the recipient mice recovered more rapidly from CCl_4_ injury compared to the CCl_4_ injured mice that did not receive AF-MSCs [74]. However, Zagoura et al. [88] compared the effects of AF-MSCs, which are hepatic progenitor-like (HPL) cells derived from AF-MSCs and HLCs, on CCl_4_-injured livers. HPL cell transplantation had a greater therapeutic effect than AF-MSCs. In contrast, the HLCs failed to engraft and contribute to recovery.

## 5. The Paracrine Effects of Adult Somatic Stem Cells and Extraembryonic Stem Cells

Considerable researches have been performed on the role of MSCs in the treatment of liver diseases. The studies above mainly focus on MSCs differentiation into or fuse with hepatocytes when injected into injured liver tissues, serving as an effective resource for liver regeneration. Whether MSCs contribute to liver regeneration by transdifferentiation into liver cells or by paracrine effects has been ongoing discussions. MSCs treatment could produce a series of cytokines and signal molecules, such as epidermal growth factor, HGF, IL-6, TNF-a, IL-10, and IL-1 receptor antagonists, relevant to cell proliferation, angiogenesis, and anti-inflammatory responses [89]. However, MSCs can not only promote anti-inflammatory signals, but also secrete proinflammatory cytokines such as transforming growth factor *β*1 and TGF-*β*3, monocyte chemoattractant protein 1, macrophage inflammatory protein-1*α* and macrophage inflammatory protein-1*β*, or monokine [90]. Furthermore, they were also shown to reduce the proliferation of stellate cells and synthesize collagen type I through the secretion of TNF-*α* [91] and to promote hepatic stellate cell apoptosis through the secretion of nerve growth factor [92]. Higashiyama et al. suggested that MSCs mediate an antifibrotic effect through the expression of matrix metalloproteinase 9 that degrades the extracellular matrix [93]. After BMMSCs treatment, upregulation of fibrinogen-like-protein 1 expression, induction of signal transducer, and activator of transcription 3 would reduce hepatocyte apoptosis and enhance liver regeneration. At the same time, Bcl2 expression was increased, whilst the protein expression of Bax was reduced [94]. During the period after ischemia reperfusion, IL-10 was proved to rescue the liver grafts by its anti-inflammatory properties, through inhibition of allograft inflammatory factor 1 mediated proinflammatory and proapoptotic activities of the macrophages [95]. In addition, the activation of early proliferative response pathways of JNK/c-Jun, CyD1, and NF-kB would be the major mechanism for resisting ischemia reperfusion injury, promoting liver regeneration and stimulated allograft regeneration in small-for-size liver transplantation [96]. ADMSCs did not differentiate into hepatocytes after engrafting to livers within 3 days, but both concentrated serum-free ADMSCs conditional media and ADMSCs lysate demonstrated obvious improvement in terms of high survival rates of ALF rats [97]. UCMSCs secrete multiple cellular factors to stimulate host hepatocyte proliferation via a paracrine mechanism, which can promote the recovery of the host's liver [98]; they are able to reduce inflammatory agents and elevate the serum levels of HGF [99].

Meanwhile, conditioned medium (CM) from cultured MSCs can also inhibit hepatocellular apoptosis and stimulate liver regeneration by upregulating hepatic gene expression of cytokines and growth factors. Indeed, Parekkadan et al. [100] showed that the administration of MSC-derived molecules, either by a bolus of CM or by extracorporeal support using a bioreactor, significantly improved short-term survival in a D-galactosamine-induced rat model of ALF. Another study [101] confirmed that systemic infusion of CM prevented the release of liver injury biomarkers and provided a significant survival benefit. What is more is that increased expression of VEGF and matrix metallopeptidase 9 in the grafts and induced Akt and ERK phosphorylation were observed after CM therapy [102]. ADMSCs and ADMSCs secretome infusions [103] both alleviated liver damage and improved the liver microenvironment after hepatic ischemia/reperfusion injury, which indicated that ADMSCs work* in vivo* through a paracrine effect. In addition, the AF-MSCs derived HPL-CM [88] was found to be more efficient than CM derived from AF-MSCs in treating the liver disease. Proteome profile analysis of HPL-CM indicated the presence of anti-inflammatory factors such as IL-10, IL-1 receptor antagonists, IL-13, and IL-27, which may have induced liver recovery. Blocking studies of IL-10 secretion from HPL cells confirmed the therapeutic significance of this cytokine in an ALF mouse model.

Genetically modified MSCs implanted in the liver graft may offer a novel approach to promoting liver regeneration. HGF [104] and C-X-C chemokine receptor type 4 (CXCR4) overexpression [105] enhanced the mobilization and engraftment of MSCs into small-for-size liver grafts, in which these cells promoted the early regeneration of the remnant liver by a paracrine mechanism. Ma et al. [106] demonstrated that CXCR4-MSCs or normal MSCs exhibited a paracrine effect through secreting HGF and VEGF, but genetically modified MSCs expressing CXCR4 showed greater colonization and conferred better functional recovery in damaged liver. Targeting androgen receptor [107] in the BMMSCs improved their self-renewal and migration potentials and increased paracrine effects to exert anti-inflammatory and antifibrotic actions to enhance liver repair. However, knocking out androgen receptor in BMMSCs led to improved self-renewal and migration by alteration of the signaling of epidermal growth factor receptor and matrix metalloproteinase 9 and resulted in suppression of infiltrating macrophages and hepatic stellate cell activation through modulation of IL1 receptor/IL1 receptor antagonists signaling. Zhang et al. [108] infected ADMSCs with a lentivirus encoding HGF and HGF short hairpin RNA. The HGF overexpressing ADMSCs ameliorated radiation-induced liver fibrosis through the downregulation of *α*-SMA and fibronectin, and an enhanced level of hepatocyte regeneration was observed simultaneously. Another study investigating epigenetic modification demonstrated that AF-MSCs overexpressing IL-1 receptor antagonists [109] prevented liver failure and reduced mortality in rats with fulminant hepatic failure, improved liver function, and increased survival rates after the rats were injected with these cells.

The MSCs and paracrine effects of MSCs may take synergistic effect for* in vivo* liver regeneration. Regarding the mechanism of the therapeutic effect of MSCs, the paracrine effect of MSCs should be more thoroughly addressed. Further studies* in vitro* and* in vivo* are needed to achieve a better understanding of the paracrine effects of MSCs.

## 6. Conclusions

MSCs have attracted attention for regenerative medicine, especially for treating end stage liver diseases. As mentioned in this review,* in vitro* studies have shown that, under proper stimulation, MSCs are capable of acquiring hepatic characteristics including polygonal morphology, ALB secretion, and the expression of several CYP enzymes. Additionally, the expression of these markers is sufficient to support hepatic functions. Furthermore, transplantation of undifferentiated MSCs and differentiated MSCs seems to ameliorate liver injury and improves liver functions. However, before clinical applications can be considered, there are still many gaps in our understanding of basic stem cell biology, such as the characterization of MSCs, the mechanisms of their differentiation, and the optimum culturing conditions for proliferation and the characteristics, as well as maintenance, of the differentiated phenotype. Additionally, novel sources of MSCs for clinical use need to be discovered, and signal patterns must be determined for* in vitro* and* in vivo* hepatic differentiation. Then, we can artificially regulate the differentiation step to induce mature hepatocytes by imitating the* in vivo* environment for various types of MSCs. Ideally, cells for liver therapies should proliferate extensively* in vitro* and then differentiate into hepatocytes that are immune compatible and are able to reconstitute liver tissue when transplanted* in vivo*.

All in all, MSCs therapy of liver diseases is promising, and further evaluation in large randomized and controlled clinical trials with extended follow-ups periods to make analytical comparisons between the studies is required for safe clinical use. Thus, generating mature and functional HLCs* in vitro* and* in vivo* can effectively replace liver transplantation and primary hepatocyte transplantation for promoting liver regeneration.

## Abbreviations

ALF: Acute liver failure
HSCs: Hepatic stem cells
ESCs: Embryonic stem cells
iPSCs: Induced pluripotent stem cells
MSCs: Mesenchymal stem cells
BMMSCs: Bone marrow mesenchymal stem cells
ADMSCs: Adipose derived mesenchymal stem cells
MenSCs: Menstrual blood stem cells
FGF: Fibroblast growth factor
HGF: Hepatocyte growth factor
HNF: Hepatocyte nuclear factor
AFP: Alpha fetoprotein
HLCs: Hepatocyte-like cells
IL: Interleukin
VEGF: Vascular endothelial growth factor
ALB: Albumin
CYP: Cytochrome
CK: Cytokeratin
CCl_4_: Carbon tetrachloride
TNF: Tumor necrosis factor
EGFP: Enhanced green fluorescent protein
PDMSCs: Placental derived mesenchymal stem cells
UCMSCs: Umbilical cord mesenchymal stem cells
UCBMSC: Umbilical cord blood mesenchymal stem cells
CV-MSCs: Chorionic villi mesenchymal stem cells
AE-MSCs: Amnion mesenchymal stem cells
CP-MSCs: Chorionic plate mesenchymal stem cells
WJ-MSCs: Wharton's jelly MSCs
AF-MSCs: Amniotic fluid mesenchymal stem cells
HPL: Hepatic progenitor-like
TGF: Transforming growth factor
CM: Conditioned medium
CXCR4: C-X-C chemokine receptor type 4.
